# Analyses of changes in myocardial long non-coding RNA and mRNA profiles after severe hemorrhagic shock and resuscitation via RNA sequencing in a rat model

**DOI:** 10.1186/s12867-018-0113-8

**Published:** 2018-11-01

**Authors:** Lin Lin, Zhengfei Yang, Guanghui Zheng, Yongxun Zhuansun, Yue Wang, Jianguo Li, Rui Chen, Wanchun Tang

**Affiliations:** 1 Sun Yat-sen Memorial Hospital, Sun Yat-sen University, 107 Yan Jiang Xi Road, Guangzhou, 510120 China; 2 Weil Institute of Emergency and Critical Care Research, School of Medicine, Virginia Commonwealth University, Richmond, VA USA; 3 Department of Emergency Medicine, Virginia Commonwealth University, Richmond, VA USA

**Keywords:** Hemorrhagic shock, Ischemia reperfusion injury, Long non-coding RNA, STAT3, High-throughput RNA sequencing

## Abstract

**Background:**

Ischemia–reperfusion injury has been proven to induce organ dysfunction and death, although the mechanism is not fully understood. Long non-coding RNAs (lncRNAs) have drawn wide attention with their important roles in the gene expression of some biological processes and diseases, including myocardial ischemia–reperfusion (I/R) injury. In this paper, a total of 26 Sprague–Dawley (SD) rats were randomized into two groups: sham and ischemia–reperfusion (I/R) injury. Hemorrhagic shock was induced by removing 45% of the estimated total blood volume followed by reinfusion of shed blood. High-throughput RNA sequencing was used to analyze differentially expressed (DE) lncRNAs and messenger RNAs (mRNAs) in the heart tissue 4 h after reperfusion. Myocardial function was also evaluated.

**Results:**

After resuscitation, the decline of myocardial function of shocked animals, expressed by cardiac output, ejection fraction, and myocardial performance index (MPI), was significant (p < 0.05). DE lncRNAs and mRNAs were identified by absolute value of fold change ≥ 2 and the false discovery rate ≤ 0.001. In rats from the I/R injury group, 851 lncRNAs and 1015 mRNAs were significantly up-regulated while 1533 lncRNAs and 1702 m RNAs were significantly down-regulated when compared to the sham group. Among the DE lncRNAs, we found 12 location-associated with some known apoptosis-related protein-coding genes which were up-regulated or down-regulated accordingly, including STAT3 and Il1r1. Real time PCR assays confirmed that the expression levels of five location-associated lncRNAs (NONRATT006032.2, NONRATT006033.2, NONRATT006034.2, NONRATT006035.2 and NONRATT029969.2) and their location-associated mRNAs (STAT3 and Il1r1) in the rats from the I/R injury group were all significantly up-regulated versus the sham group.

**Conclusions:**

The DE lncRNAs (NONRATT006032.2, NONRATT006033.2, NONRATT006034.2 and NONRATT006035.2) could be compatible with their role in myocardial protection by stimulating their co-located gene (STAT3) after hemorrhagic shock and resuscitation. The final prognosis of I/R injury might be regulated by different genes, which is regarded as a complex network.

**Electronic supplementary material:**

The online version of this article (10.1186/s12867-018-0113-8) contains supplementary material, which is available to authorized users.

## Background

Severe hemorrhagic shock, which is a major cause of morbidity and mortality for patients suffering from trauma, visceral hemorrhage, or major surgery, can lead to myocardial dysfunction [1–4]. The immediate presence of adequate perfusion of the tissue (reperfusion) in patients of hemorrhagic shock is the fundamental goal of acute resuscitation, however it may result in an injury that potentially induces organ dysfunction and death, known as ischemia–reperfusion (I/R) injury [5]. Suggested mechanisms of I/R injury include the generation of reactive oxygen species, intracellular Ca^2+^ overload, the increase of proinflammatory cytokines, and mitochondrial dysfunction, which subsequently results in apoptosis [6–8]. Although defining its complex pathophysiological process remains a challenge, ascertaining how to relieve I/R injury is of great importance. To that end, thorough knowledge of the genetic basis of I/R injury may provide therapeutic targets and tools.

Long non-coding RNAs (lncRNAs) are a family of RNA molecules (containing more than 200 nucleotides) which do not encode proteins, but functionally participate in many processes controlling gene expression and cell differentiation [9]. In recent years, lncRNAs have drawn wide attention with their critical roles in gene expression of various biological processes and diseases, including Parkinson’s disease [10], liver disease [11], gynecological diseases [12] and tumors [13, 14]. They serve as biomarkers, prognostic assessments, and even therapeutic targets. Wang et al. [15] reported that an lncRNA named necrosis-related factor (NRF) reduced myocardial infarct size upon I/R injury in the animal model, with miR-873 as one of its targets suppressing the expression of RIPK1 (receptor-interacting serine/threonine-protein kinase 1)/RIPK3 (receptor-interacting serine/threonine-protein kinase 3) and decreasing death of cardiac cells. However, the operating function of lncRNAs may be a complex network involving different facets of cell biology. More lncRNAs and their effects in the mechanism of I/R injury remain to be identified.

In our previous study [16], we preliminarily confirmed the correlation between the phosphorylation of protein STAT3 and the myocardial protective mechanism after myocardial I/R injury. In this study, we identified changes in the expressions of mRNAs and lncRNAs in myocardia after severe hemorrhagic shock and resuscitation models compared with sham rats using RNA sequencing techniques, aiming to explore the upstream regulator of protein STAT3 for cardioprotective effects. Our findings predict the regulatory role of lncRNAs. It could lead to further research to explore therapeutic targets for cardioprotection in I/R injuries induced by hemorrhagic shock and resuscitation.

## Results

### Model identification of I/R injury caused by hemorrhagic shock and resuscitation

**Fig. 1:**
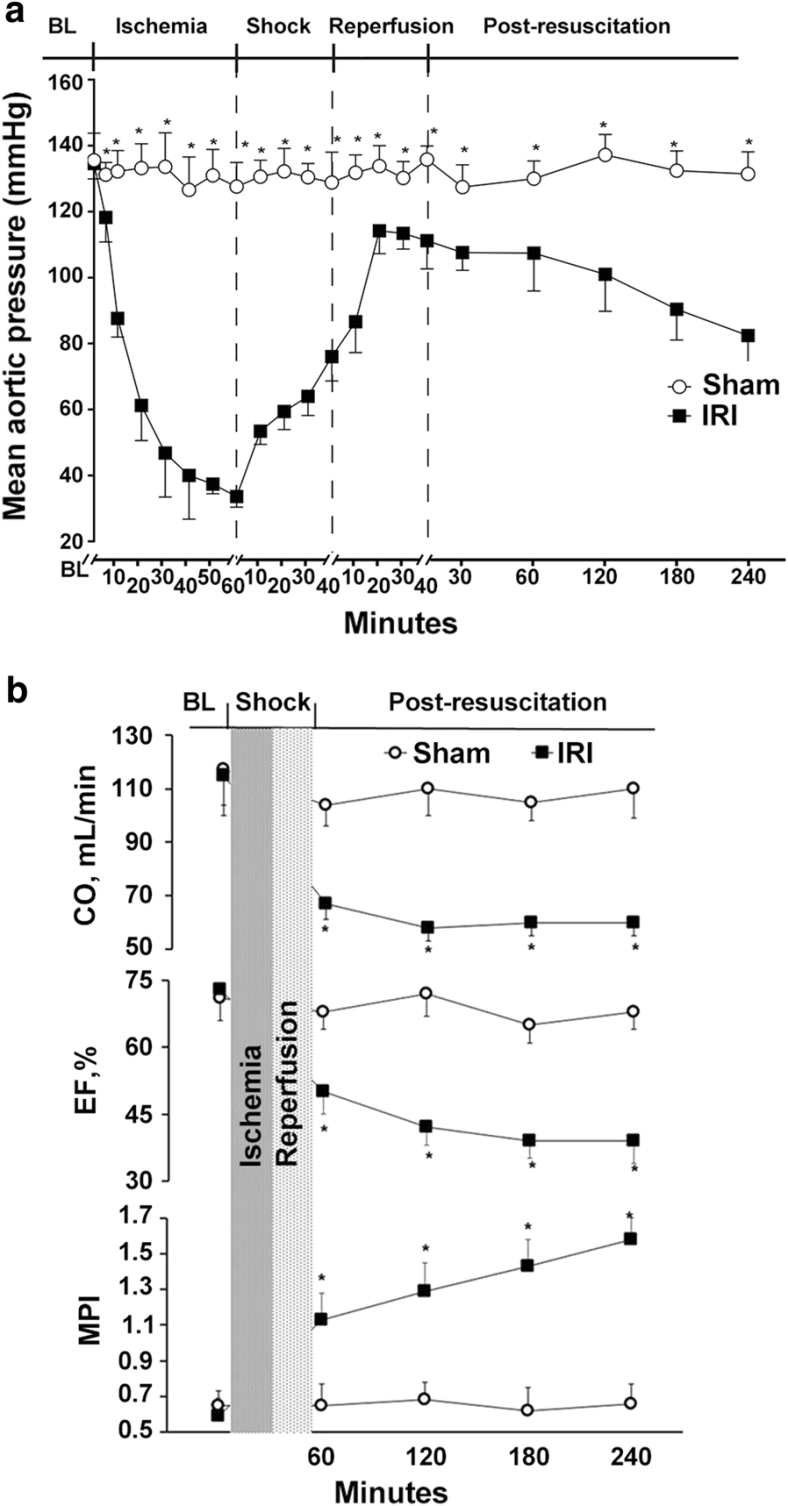
Changes of **a** mean aortic pressure and **b** myocardial function during and after hemorrhagic shock and resuscitation. *IRI* ischemia reperfusion injury, *CO* cardiac output, *EF* ejection fraction, *MPI* myocardial performance index. *p < 0.05 versus sham group

Between the I/R injury and sham groups, there was no difference in the baseline characteristics (Table 1). In the hemorrhage and shock phases, mean arterial pressure (MAP) of the I/R injury group was significantly lower compared to the sham group (p < 0.05). In the resuscitation phase, MAP of the I/R injury group rapidly returned to normal, nearly matching the baseline (Fig. 1a). After resuscitation, the CO and EF levels of the I/R injury group had significantly decreased compared to the sham group and baseline value (p < 0.05). Meanwhile, the MPI of the I/R injury group showed a significant raise compared to the sham group since the resuscitation phase (p < 0.05, Fig. 1b).

**Table 1:** Baseline characteristics of two groups

Variables	Sham	IRI
Body weight (g)	405 ± 10	395 ± 15
Heart rate (beats/min)	388 ± 32	379 ± 25
Mean artery pressure (mmHg)	131 ± 12	135 ± 10
Rectal temperature (°C)	37.3 ± 0.4	36.9 ± 0.6
ETCO_2_ (mmHg)	35.9 ± 3.2	37.1 ± 4.9
Arterial lactate (mmol/l)	1.2 ± 0.2	0.9 ± 0.3
Arterial pH	7.45 ± 0.06	7.48 ± 0.04

Values are presented as mean ± SD

*IRI* ischemia–reperfusion injury, *ETCO*_*2*_ end-tidal CO_2_

### DE lncRNAs and mRNAs

We examined all significantly different expression levels of the transcripts in the heart tissue of I/R injury rats as previously described [17]. Differentially expressed lncRNAs and mRNAs were identified by the absolute value of fold change bigger than 2 and the FDR < 0.001. The total reads (mRNAs and lncRNAs) of the two samples were 163, 128, 480 and 84, 501, 062, respectively, while the clean bases of the two samples were 24.47G and 12.68G. The raw data in this study are available in the NCBI SRA database.

**Fig. 2:**
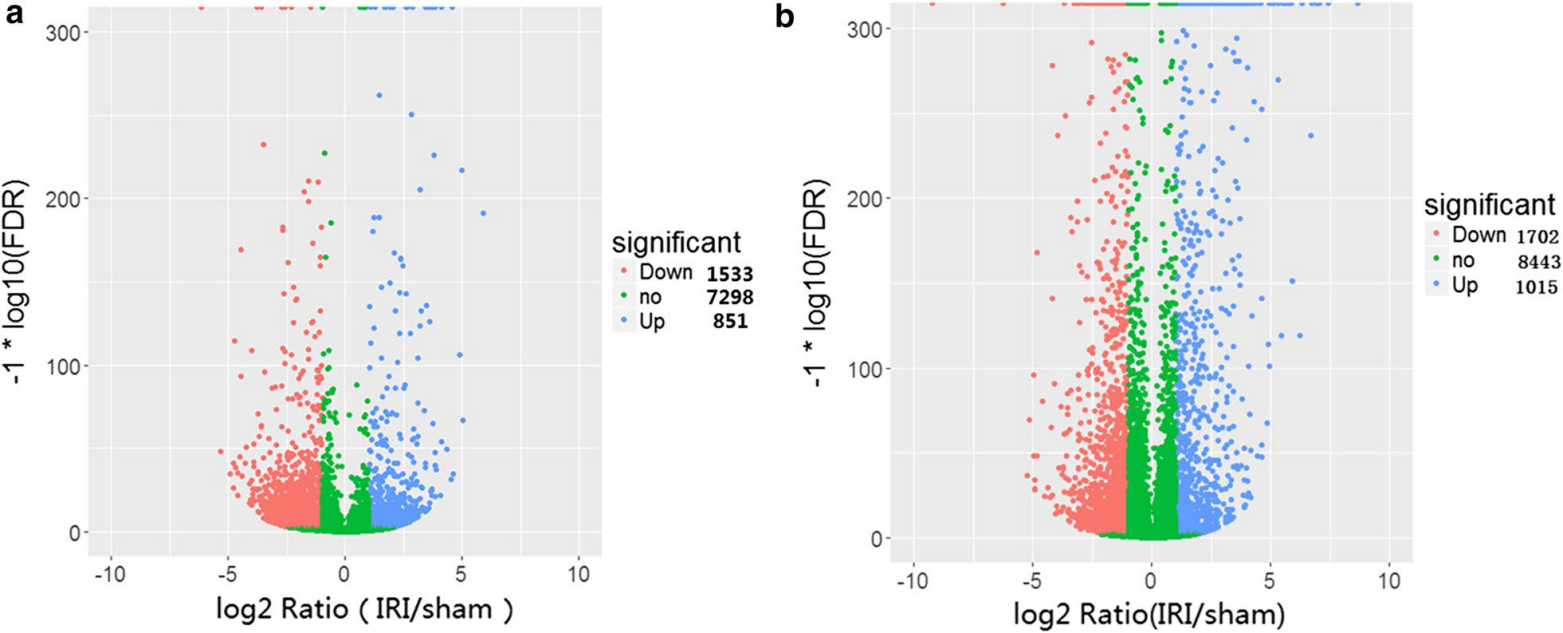
The changes in expression profiling of lncRNAs (**a**) and mRNAs (**b**) in cardiac tissue of ischemia reperfusion injury in rats induced by hemorrhagic shock and resuscitation compared with sham group

Our data revealed that 851 lncRNAs were up-regulated in rats from the I/R injury group, and 1533 lncRNAs were down-regulated when compared to the sham group (Fig. 2a). Meanwhile, 1015 mRNAs were significantly up-regulated and 1702 m RNAs were significantly down-regulated versus the sham group (Fig. 2b). The top 5 up-regulated and down-regulated lncRNAs and mRNAs are listed in Tables 2 and 3.

**Table 2:** The detail information of the top 5 up-regulated and 5 down-regulated lncRNAs

geneID	Gene length	IRI (RP KM)	Sham (RP KM)	log2 ratio (IRI/sham)	Regulation (IRI/sham)	p-value	FDR
NONRATT017111.2	807	51.98819551	0.860104443	5.917528414	Up	0	0
NONRATT017580.2	614	25.69424929	0.791324104	5.021032989	Up	0	0
NONRATT007613.2	687	74.09044896	2.323784361	4.994739473	Up	0	0
NONRATT013472.2	342	73.70572335	2.435453632	4.919514229	Up	0	0
NONRATT020494.2	976	8.845908389	0.355586212	4.636739013	Up	0	0
NONRATT012903.2	1379	0.754310054	54.51159832	− 6.175261756	Down	0	0
NONRATT013793.2	2141	0.097168946	3.922775269	− 5.33523556	Down	0	0
NONRATT009047.2	365	0.569969077	17.4952313	− 4.939934279	Down	0	0
NONRATT001411.2	1652	0.104942854	2.941119852	− 4.808689746	Down	0	0
NONRATT031486.1	2039	0.136039701	3.778596157	− 4.79575069	Down	0	0

*IRI* ischemia–reperfusion injury, *FDR* false discovery rate

**Table 3:** The detail information of the top 5 up-regulated and 5 down-regulated mRNAs

geneID	Gene length	IRI (RP KM)	Sham (RP KM)	log2 ratio (IRI/sham)	Regulation (IRI/sham)	p-value	FDR
Selp	3168	88.69664	0.219099	8.66115554	Up	0	0
Cxcl10	1134	849.7054	5.019096	7.403391386	Up	0	0
Ubd	671	139.3126	1.034433	7.073341863	Up	0	0
Ifit1	2040	302.5909	2.483805	6.928673368	Up	0	0
Cxcl9	554	158.0944	1.503475	6.716341599	Up	0	0
Spta1	7945	0.122196	73.97081	− 9.241615075	Down	0	0
Aplnr	3646	0.98903	75.02647	− 6.24524114	Down	0	0
Haus8	1447	0.11981	4.50904	− 5.233995581	Down	0	0
Irx5	1455	0.238303	8.491448	− 5.15514016	Down	0	0
Foxs1	1270	0.218413	6.941043	− 4.990019511	Down	0	0

*IRI* ischemia–reperfusion injury, *FDR* false discovery rate

### DE lncRNA target mRNAs

LncRNAs have been shown to function as regulators of adjacent protein-coding genes through RNA–protein interactions [18, 19]. In our study, lncRNAs located within 5 kb of known protein-coding genes, or those contained in the introns of genes, were considered to be location-associated lncRNAs. Among the DE lncRNAs, we found 12 of them location-associated with some apoptosis—related protein-coding genes (Table 4). For instance, four lncRNAs named NONRATT006032.2, NONRATT006033.2, NONRATT006034.2, and NONRATT006035.2 were location-associated with STAT3; NONRATT029969.2 was location-associated with Il1r1. STAT3 codes STAT3 proteins and our previous research showed they were involved in the mechanism of cardio-protection. Consistent with the lncRNAs, expressions of the location-associated mRNAs were up-regulated or down-regulated in I/R injury group. On these grounds we affirm that lncRNAs could participate in expressions of co-located protein-coding genes. The sequence read data of the research can be obtained from the NCBI database with the accession code: SRP164364.

**Table 4:** Location-associated lncRNAs and corresponding protein-coding genes

Gene	Position	IRI RPKM	Sham RPKM	Fold change	Regu-lation	Gene	Position	IRI RPKM	Sham RPKM	Fold change	Regulation
NONRATT006032.2	chr10:88791856-88796598	7.831	1.444	5.424	Up	Stat3	chr10:88790406-88842233	273.503	58.99	4.667	Up
NONRATT006033.2	chr10:88792525-88797203	3.650	0.685	5.328	Up						
NONRATT006034.2	chr10:88793064-88796762	26.141	3.089	8.463	Up						
NONRATT006035.2	chr10:88809397-88810399	5.958	2.053	2.902	Up						
NONRATT009101.2	chr13:105055964-105056435	5.069	0.735	6.894	Up	Tgfb2	chr13:105041053-105140780	6.410	3.680	1.742	Up
NONRATT009102.2	chr13:105137574-105138070	4.744	0.698	6.794	Up						
NONRATT011994.2	chr16:50029795-50031018	4.221	1.928	2.189	Up	Tlr3	chr16:50016857-50030314	14.447	6.461	2.236	Up
NONRATT020804.2	chr4:146777105-146777750	3.006	13.001	0.231	Down	Atg7	chr4:146598416-146776407	9.284	7.338	1.265	Up
NONRATT021717.2	chr4:146778556-146779157	4.377	12.106	0.362	Down						
NONRATT026198.2	chr7:126685426-126687282	19.157	51.507	0.372	Down	Ppara	chr7:126619196-126681752	20.588	65.117	0.316	Down
NONRATT029969.2	chr9:47035533-47036225	119.680	31.350	3.818	Up	Il1r1	chr9:46997798-47035275	68.896	31.162	2.211	Up
NONRATT029995.2	chr9:54304608-54308682	8.083	0.973	8.310	Up	Stat1	chr9:54287540-54327958	124.250	67.559	1.839	Up

*IRI* ischemia–reperfusion injury

### Real-time quantitative PCR

**Fig. 3:**
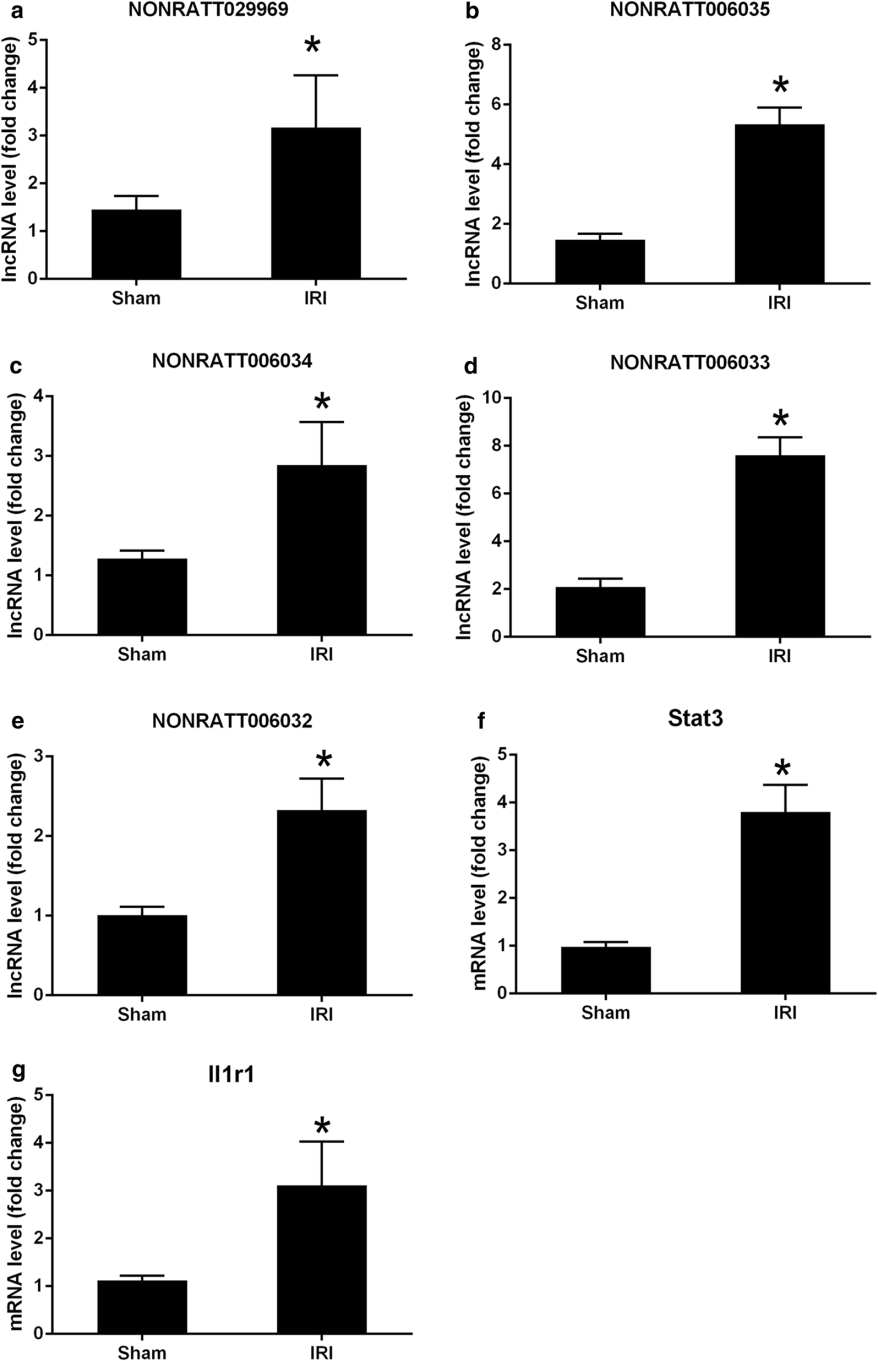
QPCR validations of five lncRNAs and two mRNAs in cardiac tissue of ischemia reperfusion injury in rats induced by hemorrhagic shock and resuscitation. The expressions of lncRNAs (**a**–**e**) and mRNAs (**f**, **g**) were significantly up-regulated 4 h after resuscitation compared with sham group. One-way ANOVA followed by Tukey’s multiple comparison test. *p < 0.05 versus sham group

To validate the accuracy of lncRNA and mRNA expression profiles determined by next generation RNA sequencing, several specific location-associated lncRNAs and mRNAs in the heart segments of intact rats were confirmed by real-time PCR assay. The expression levels of five location-associated lncRNAs in the I/R injury group, NONRATT006032.2, NONRATT006033.2, NONRATT006034.2, NONRATT006035.2 and NONRATT029969.2, were all significantly up-regulated versus the sham group (Fig. 3a–e). The expression levels of two location-associated mRNAs in the I/R injury group, STAT3 and Il1r1, were also significantly enhanced compared to the sham group (Fig. 3f, g).

## Discussion

Several studies have highlighted that I/R injury, ultimately leading to heart failure, is a major cause of morbidity and mortality [20, 21]. However, the molecular mechanism is not fully understood. In the present study, which is the first of its kind, we utilized next-generation RNA sequencing technology and bioinformatics analysis to investigate whole mRNA and lncRNA profiles of heart tissue in rat models suffering from I/R injury caused by severe hemorrhagic shock. The results of our research indicated differential expression of mRNAs and lncRNAs in the cardiomyocytes between the I/R injury group and sham group after hemorrhagic shock and resuscitation. In the I/R injury group 851 up-regulated and 1533 down-regulated lncRNAs were deemed to be involved in the I/R injury. Meanwhile, 1015 up-regulated transcriptions and 1702 down-regulated transcriptions in the I/R injury group were found to be DE mRNAs. To further validate the reliability of the sequencing results, expressions of several DE lncRNAs and mRNAs were verified by qRT-PCR.

A family of RNA, which had long been considered as a platform for protein production until recently, was reported to play a major role in pathophysiology of human diseases including complex heart diseases [7, 22, 23]. Meanwhile, their potential roles in I/R injuries were also proven [20, 21]. However, myocardial lncRNA expression and how it functions in hemorrhagic shock induced I/R injuries haven’t yet been fully explored. Liu et al. [24] investigated lncRNA expression in the infarct region of mice after myocardial ischemia by RNA-sequencing analysis. They reported 31,423 arrayed lncRNAs, of which 151 were aberrantly expressed (64 up-regulated and 87 down-regulated). In contrast, our research found a remarkably greater number of dysregulated lncRNAs. There are several possible reasons for the divergence. Firstly, different species of animal were used in the two studies, and the gene expression varies between species. Secondly, we analyzed DE profiles by high throughput RNA-sequencing techniques rather than gene microarray. The former could detect a great quantity of novel transcriptions more comprehensively [25]. Thirdly, our research was based on I/R models induced by bleeding (removing almost half estimated total blood volume) followed by resuscitation of shed blood, and the diffusely ischemic heart tissue was used instead of the focal infarct sections. The Liu’s I/R models were performed via occlusion followed by reperfusion of the left anterior coronary artery, which led to myocardial infarction. Our animal models simulated more physiopathology processes found in severe hemorrhagic shock. The significant difference of the DE lncRNA numbers between the two studies indicated that aberrant expression of lncRNA may has tissue specificity. Finally, the two studies screened DE lncRNAs at different time points (2 h and 4 h after I/R injury respectively), in which durations of ischemia were also different (45 min and 60 min respectively). This discrepancy uncovered that the expression of lncRNAs involved in I/R injury might be correlated with the degree of injury. Moreover, how to select the optimal timing for treatment of I/R injury is also an interesting topic, which is absolutely worth further study.

The balance between cell survival and death is generally regarded as the prime determinant of prognosis for ischemic injury [26]. As a main type of programmed cell death, apoptosis is proven to be crucial to an I/R injury [27, 28], which is regulated by several signaling pathways including the janus kinase (JAK)/signal transducer and activator of transcription (STAT) pathway. Evidence has demonstrated that the activation of STAT3 limits apoptosis in rat models of myocardial infarction [29], while others have highlighted the importance of JAK2-STAT3 activation in apoptosis of post-ischemic damage [30].

The JAK/STAT pathway is activated after the binding of a ligand to the receptor in the plasma membrane. The ligand phosphorylates the receptor by activating JAK kinases then binds cytosolic STAT proteins to it. The STAT proteins are phosphorylated and translocated into the nucleus to regulate the transcription of target DNA sequences [31, 32]. Actually about 10 years ago, Oshima [33] reported that cardiac-specific transgenic mice expressing constitutively active STAT3 exhibited resistance against I/R injury, compared with non-transgenic littermates. The data of Xie et al. [34] revealed that elevated expression of JAK2/STAT3 mRNA and an activated JAK2/STAT3 signaling pathway acted as a cardioprotective factor in I/R injury mouse models. Our group recently reported the existence of a cardioprotective process called remote ischemic post-conditioning (RIPostC) in rats [16]: the RIPostC group demonstrated attenuated myocardial injuries, increased survival rate and enhanced phosphorylation of STAT3 4 h after reperfusion. We verified that RIPostC protected cardiomyocytes partially by phosphorylating STAT3 through the SAFE pathway. This work result was consistent with the hypothesis that lncRNAs function as an upstream contributor of the JAK/STAT signaling pathway to confer cardio-protection in an I/R injury.

In the present study, we identified increased levels of 4 relevant lncRNAs (NONRATT006032.2, NONRATT006033.2, NONRATT006034.2 and NONRATT006035.2) in myocardial cells after an I/R injury. Meanwhile, a significant increase in the abundance of STAT3, predicted to be lncRNA co-located mRNA, was found. It is reported that the positions of about 65% lncRNAs are within 10 kb of known protein-coding genes, which may be involved in cis-acting or trans-regulatory relationships with their neighbor protein-coding genes [18, 19]. This means lncRNAs may modulate the expression patterns of these location-associated genes. We concluded that the aforementioned 4 enhanced lncRNAs up-regulated the expression of location-associated mRNA STAT3. They might proceed to stimulate the expression of STAT3 protein which was verified to relate to the anti-apoptotic mechanism after I/R injury.

However, the I/R injury group in our research showed a significant decrease rather than improvement in myocardial function despite cardio-protection of STAT3 during ischemia and reperfusion. Not surprisingly, we expect that other of the DE lncRNAs involved in pathogenesis of I/R injury may conspire to cause the decline of myocardial function. For instance, we confirmed that NONRATT029969.2 co-located with and significantly up-regulated its adjacent protein-coding gene IL1R1 by both high-throughput sequencing and qPCR. Stimulation of the interleukin 1 receptor (IL-1R) leads to the recruitment of adaptor proteins such as myeloid differentiation factor 88 (MyD88), which triggers large-scale downstream signaling cascades and production of proinflammatory cytokines [35, 36]. IL-1R antagonist was reported to have protective effects against I/R injury by attenuating the inflammatory response, which was associated with decreased apoptosis [37, 38].

Our analyses may indicate the complex network relationships among lncRNAs, mRNAs and involved proteins after I/R injury caused by severe hemorrhagic shock. Moreover, certain lncRNAs are likely to modulate their mRNA targets, not only neighbor protein-coding genes, by interacting with miRNAs and inhibiting the ability of them. This suggested a novel perspective to screen and predict target mRNAs involved in physiopathology mechanisms of I/R injury in our future research.

There are several limitations in our study. Firstly, due to the limited funds and resources, we chose to validate only 5 lncRNAs and 2 mRNAs by PCR, which might be involved in our previous study results, remaining other DE mRNAs to be analyzed in our further research. Secondly, knockdown of the 4 lncRNAs (NONRATT006032.2, NONRATT006033.2, NONRATT006034.2 and NONRATT006035.2) was not performed. Whether the lncRNA knockdown would increase cell apoptosis and reverse cardioprotective effects after hemorrhagic shock remains unknown. Thirdly, in this study we tested only three samples from the I/R injury and sham groups for RNA sequencing. More samples will be needed to verify the results in future research. Finally, the number of time points for sample collecting was relatively limited. Further studies should detect the lncRNA expression levels at more time points to determine if the regulation effect of lncRNAs is associated with length of ischemia and reperfusion time.

## Conclusions

Our research provides evidence of location-associated modulation roles of lncRNAs (NONRATT006032.2, NONRATT006033.2, NONRATT006034.2 and NONRATT006035.2) in I/R injury rat models caused by hemorrhagic shock by targeting their co-located gene (STAT3). We predict that they may confer myocardial protection by stimulating expressions of downstream proteins (STAT3 protein) and reducing apoptosis through activated JAK2/STAT3 pathway. However, the final prognosis of I/R injury could be regulated by different genes, which is regarded as a complex network.

## Methods

### Animals

Experiments were performed on 26 male Sprague–Dawley rats weighing between 350 and 450 g (the Experimental Animal Center of Sun Yat-sen University, Guangzhou, China). The rats were kept in a specific sterile laboratory, at a uniform temperature of 20–22 °C and on a light/dark cycle of 12 h. Our research was approved by the Institutional Animal Care and Use Committee, established in the Tang Wanchun Laboratories of Emergency Critical Care Medicine (Sun Yat-sen Memorial Hospital, Sun Yat-sen University, IACUC-2016R1604).

### Animal preparation

After fasting overnight, with the exception of free access to water, the rats were anesthetized by intraperitoneal injection of sodium pentobarbital (45 mg/kg) and then stretched supinely on a surgical board. To maintain anesthesia, an additional administration of sodium pentobarbital (10 mg/kg) was required. The trachea was orally intubated with a 14-G cannula (Abbocath-T; Abbott Hospital Products Division, North Chicago, IL) mounted on a blunt needle with a 145° angled tip. End-tidal carbon dioxide pressure (ETCO_2_) was measured by the capnometer module of a BeneView T5 patient monitor (Mindray, Shenzhen, China), and a conventional lead II electrocardiogram was continuously monitored. Spontaneous respiration of room air was maintained. The rectal temperature was kept at 37 °C ± 0.5 °C with infrared surface heating lamps.

The left femoral artery was surgically exposed, into which a polyethylene catheter (PE-50; Becton–Dickinson, Franklin Lakes, NJ) was advanced to the descending aorta to measure aortic pressure and blood gas. Another PE-50 catheter was advanced into the right atrium through the left external jugular vein to measure the right atrial pressure. Saline with 2.5 IU/ml of crystalline bovine heparin was flushed intermittently to all catheters.

### Experimental procedures

After surgical preparation, the animals were randomized into two groups: I/R injury and sham group. In the I/R injury group the animals were heparinized with 100 U/kg of bovine heparin. An estimated 45% of total blood volume, according to the blood volume of each animal (EBV = 6.12 ml/100 g BW) [16], was withdrawn over an interval of 60 min. An infusion/withdrawal dual syringe pump (LongerPump LSP01-1A, Longer Corporation, China) was used for bleeding and recovery of shed blood. Blood from the left femoral artery was allowed to flow into a sterile 20 ml syringe. The reinfusion of shed blood was performed 40 min after the completion of bleeding, over the ensuing 40 min. In the sham group, the procedures of animal preparation rather than bleeding were performed. All the animals were then monitored for an additional 4 h. ETCO_2_, electrocardiography, temperature, and aortic pressures were recorded at intervals of 5-min during hemorrhage and 15-min thereafter. Myocardial functions were measured at baseline and hourly after the ending of reinfusion. After the 4 h period, the animals were euthanized by intraperitoneal injection of pentobarbital (150 mg/kg) for the collection of heart tissue. Three rats from both groups were randomly selected for RNA sequencing while others remained for quantitative real-time RT-PCR evaluation.

### Measurement

We used a PC-based data-acquisition system supported by WINDAQ software (DATAQ, Akron, OH) to make continuous records of ETCO_2_ levels, electrocardiogram output, and aortic and right atrial pressures. Myocardial function was non-invasively measured with an Ultrasonix ultrasound system (Model130-4311, Ultrasonix Medical Corpration, Canada) by two separate units, covering cardiac output (CO), ejection fraction (EF), and myocardial performance index (MPI). CO and EF served as indexes of myocardial contractile function and MPI as a marker of both systolic and diastolic function [39]. Aortic blood pH, PCO_2_, PO_2_, hemoglobin, and lactate concentrations were measured in 200 μL aliquots of blood with a Stat Profile pHOx Plus L analyzer (Model RADIOMETER ABL80FLEX; Radiometer Medical ApS, Bronshoj, Denmark).

### Tissue collection and RNA isolation

Three rats from each group were randomly selected to collect heart segments. Total RNA was extracted from the heart tissue by Trizol Reagent (TR118-500, MRC) as shown in the manufacturer’s instructions. Quantity and quality of RNA were assessed by a Nano-100 (AoSheng Biotech Co. Ltd.) while RNA integrity was confirmed by 2100 bioanalyzer (Agilent).

### Library construction and sequencing

We prepared total RNAs (1 μg per sample) as input material, removed ribosomal RNAs and constructed sequencing libraries using a VAHTS Total RNA-seq (H/M/R) Library Prep Kit from Illumina^®^ (NR603-02, Vazyme) according to the manufacturer’s recommendation. The first chain of cDNA was generated using random hexamer primers and Reverse Transcriptase. The second chain was synthesized using RNase H and DNA pol I. dNTPs with dTTP were replaced by dUTP and the reaction buffer. The 3′ ends of DNA fragments were adenylated, and an Illumina sequencing Adapter was ligated for hybridization. VAHTS DNA Clean Beads (N411-03, Vazyme) were used to purify the library fragments to select cDNA fragments of 350–400 bp. Then PCR amplification was performed to establish the complete sequence of the cDNA library. Library quality was assessed on a 2100 bioanalyzer (Agilent). The library was sequenced on a Nextseq500 platform (Illumina Company, USA), and 150 bp paired-end reads were generated. We analyzed the sequencing results and dynamically removed joint sequence fragments and low-quality segments from the 3′ end. FastQC software was used for quality control of the raw data.

### Comparison with reference genome

Reference genome sequencing alignment was performed against the whole genome sequence using TopHat software. The reference genome was Rattus norvegicus (rn6) (ftp://ftp.ensembl.org/pub/release-81/fasta/rattus_norvegicus/dna/). The known transcript information in the genome location was downloaded from Ensembl (ftp://ftp.ensembl.org/pub/release-81/gtf/rattus_norvegicus/Rattus_norvegicus.Rnor_6.0.81.abinitio.gtf.gz).

There followed a list of required parameters: mismatch of 2 bases was allowed; during each read optimal match record up to 20 bp was allowed; given the variable shear, fragment length of 25 bp was used; in each fragment mismatch of less than 2 bp was allowed; insertions and deletions were limited to 3 bp; to completely guarantee comparison, alternative splicing position mismatch was limited to 0; the allowed length of intron was 50–50,000 bp.

### Assembly of transcripts

The mapped reads of each sample were assembled by Cufflinks [40]. Considering read coverage gaps in the regions of the reference gene,we compensated the incompletely-assembled transcripts by performing reference annotation based transcripts (RABT) assembly with the reference gene annotation. The features could be missing in the sequencing data, attributed to low coverage. To avoid that, faux-reads were generated from reference transcripts. These reads and the (aligned) sequenced reads were merged for assembly.

### Quantitative real-time PCR

We performed quantitative analysis by quantitative real-time PCR to confirm the expression levels of several DE lncRNAs and their target mRNAs. Total RNA reverse transcription was performed using M-MLV Reverse Transcriptase (Promega, M1705) following the manufacturer’s instructions with either orligo (dT) primers or specific RT primers. cDNA was quantified in an Opticon real-time PCR machine (MJ Research, Waltham, MA, USA). The primers used are listed in Table 5. Each sample was run in triplicate in 20 μl reactions with 0.4 μM forward and reverse PCR primers and 10 μl of GoTaq^®^ qPCR Master Mix (Promega, A6002)in a ABI StepOne Plus real-time PCR system. The PCR cycle parameters were set as follows: initial denaturation at 95 °C for 2 min followed by 40 cycles of 95 °C for 15 s and 60 °C for 30 s. Relative expression was determined by normalization to GAPDH using the 2^−ΔΔCt^ method. The experiments were performed in triplicate.

**Table 5:** Primers designed for qRT-PCR validation of candidate lncRNAs and mRNAs

Gene	Forward primer	Reverse primer	Product length
NONRATT006032	TCTGACCTCGGAGTGTGCTA	ACAGCCATCACGGACTCAAG	267
NONRATT006033	AAATACTGCTGTGGAGCGGG	GATGCTGACGTGAAGTGTGC	247
NONRATT006034	TACCAAGCAGCAGCTGAACA	GGGGCGACAATACTTTCCGA	148
NONRATT006035	GGACTTCATGGTAGGACGGG	TGAGGCTCTCCACTCTGTGT	173
NONRATT029969	CTATGTTTGACAGCCGAGTTG	AGTCTCACAGAGGGATTATGGTT	158
Stat3	AACGACCTGCAGCAATACCA	TCCATGTCAAACGTGAGCGA	135
Il1r1	ACAGGGACTCCTGCTCTGAT	TCCCTCTCCGTAGGTCTTGG	95

### Statistical analysis

Normally distributed data were presented as mean ± SD while non-normally distributed data were presented as a median (25th, 75th percentiles). Statistical analysis was performed using a t-test between two cohorts. RT-PCR results were performed with one-way analysis of variance (ANOVA) followed by Tukey’s multiple comparison test. Data analysis and subsequent quantitative normalization were performed using GeneSpring GX v12.1 software (Agilent Technologies). Differentially expressed lncRNAs and mRNAs were identified by absolute value of fold change ≥ 2 and FDR ≤ 0.001. Statistical significance was set at a p < 0.05.

## Additional files

**Additional file 1: Table S1:** The raw data of mean arterial pressure, myocardial function and QPCR validation of sham group and I/R group.

**Additional file 2: Table S2:** The different expression levels of all lncRNAs between the sham group and I/R group.

**Additional file 3: Table S3:** The raw data of differentially expressed lncRNAs.

**Additional file 4: Table S4:** The different expression levels of all mRNAs between the sham group and I/R group.

**Additional file 5: Table S5:** The raw data of differentially expressed mRNAs.

## Abbreviations

lncRNAs: long non-coding RNAs
I/R: ischemia–reperfusion
SD: Sprague–Dawley
DE: differentially expressed
mRNAs: messenger RNAs
CO: cardiac output
EF: ejection fraction
MPI: myocardial performance index
FDR: false discovery rate
NRF: necrosis-related factor
MAP: mean arterial pressure
